# Prevalence of *Toxocara* antibodies among patients clinically suspected to have ocular toxocariasis: A retrospective descriptive study in Sri Lanka

**DOI:** 10.1186/s12886-017-0444-0

**Published:** 2017-04-24

**Authors:** Devika Iddawela, Kiruthiha Ehambaram, Pemindra Bandara

**Affiliations:** Department of Parasitology, Faculty of Medicine, University of Peradeniya, Peradeniya, Sri Lanka

**Keywords:** Toxocariasis, Ocular larva migrans, Vitritis, ELISA

## Abstract

**Background:**

Human toxocariasis, caused by *Toxocara canis*, *T. cati*, and *T. vitulorum* of dogs, cats and ruminants respectively, is recognized as an important zoonotic infection worldwide. The typical clinical syndromes of toxocariasis in humans are ocular larva migrans (OLM) and visceral larva migrans (VLM). The most commonly affected sites of OLM are the peripheral retina and/or vitreous humor. In Sri Lanka, there is a dearth of information on prevalence of ocular infection in our population. Therefore, the present study was carried out to determine the prevalence of *Toxocara* antibodies in suspected OLM patients and to describe demographic factors and clinical manifestations of seropositive patients. A total of 250 clinically suspected ocular toxocariasis cases referred by consultant eye surgeons to the Department of Parasitology, University of Peradeniya were studied between the years 1995 to April 2015.

**Methods:**

Data (age, sex, fundoscopic findings) were gathered from the referral letters. Each serum sample was subjected to *Toxocara* excretory – secretory antigen ELISA (TES - ELISA).

**Results:**

Out of the 250 cases, 155 (62%) were seropositive. The age range of the seropositive cases was 1 to 78 years with the mean age of 27 years. The highest seropositivity (25/155) was observed within the age group of 10 to 14 years. The most frequent clinical presentation of seropositive OLM cases were unilateral reduced vision and red eye. The other symptoms include tearing, photophobia and leukokoria. A high proportion of seropositive OLM cases had uveitis (34.19%) followed by reduced vision (21.94%), vitritis (12.9%) and choroiditis (7.74%). However none of these clinical manifestations were significantly associated with TES-ELISA seropositivity except vitreits (X^2^ = 8.557, *p* = 0.003).

**Conclusion:**

In conclusion, the results of this study showed high seroprevalence of toxocariasis among clinically suspected OLM cases confirming the toxoplasmic etiology. This high rate of *Toxocara* seropositivity in ocular patients should alert ophthalmologists in Sri Lanka to include toxocariasis in the differential diagnosis of ocular diseases presented with the symptoms and signs stated above.

## Background

Human toxocariasis is a zoonotic disease caused by several species of the nematode round worm *Toxocara*. *Toxocara s*pp. are common intestinal round worms parasitizing a wide range of domestic, agricultural, and wild animals. The species include *T. canis* of dogs and wild canids, *T. cati* of cats, *T. leonina* found in both cats and dogs and *T. vitulorum* of ruminants. The disease is most frequently caused due to *T. canis* [1]. The definitive hosts of these parasites include the domestic and peridomestic cats and dogs, particularly puppies that have been infected transplacentally [1, 2]. The adult worms in the intestines of canines and felids lay eggs that are shed along with faeces into the environment. *Toxocara* eggs, when voided by dogs are unembryonated, and need 10–14 days to develop into infective larvae [3]. Therefore, contact with eggs in soil is a very important risk factor in the transmission of toxocariasis to humans. *Toxocara* infection has been associated with pica and pet ownership [4, 5]. Age, sex, geographical location, and poor socioeconomical status are other risk factors for acquiring the disease [6, 7]. The clinical spectrum of human toxocariasis ranges from asymptomatic cases to systemic infections. The recognized clinical manifestations include classic and incomplete visceral toxocarisis (VLM), ocular toxocarisis (OLM), neurological toxocarisis (NLM), covert toxocariasis and asymptomatic toxocarisis [8].

Ocular larva migrans was first recognized by Wilder (1950) when 24 cases in which eyes enucleated for suspected retinoblastoma were found to have nematodes [1]. *Toxocara* larvae are capable of invading almost all the structures of the eye; granuloma either in the posterior pole or in the periphery of the eye has been identified as the most common clinical presentation of OLM [9–11]. The consequences of fibrosis were the most important causes of visual loss in more chronic lesions [11]. Other less common causes of visual loss in OLM include hypopyon, vitreous abscess, optic neuritis and keratitis [1, 9]. Eosinophilia which is both pronounced and persistent with VLM is virtually absent with OLM [12]. Toddlers and teenagers are the most common victims of this disease and if left untreated it may result in permanent loss of vision in the affected eye [13, 14]. OLM is typically characterized by unilateral vision impairment which may be accompanied less frequently with strabismus. Critical infection leads to invasion of the retina which leads to granuloma formation either peripherally or in the posterior pole. The granuloma drags the retina and leads to distortions, heteropia or detachment of the macula. Depending on the region of infection in the eye, the patient may have minor visual impairment or blindness. Other clinical manifestations of OLM include diffuse endophthalmitis, papillitis, or secondary glaucoma [1].

Toxocariasis represents an ‘ongoing and poorly recognized parasitic infection’ in many developing countries including Sri Lanka [15]. The first seroepidemiological study on toxocariasis in Sri Lankan population was conducted in 2003 on 1020 children in the age group 1–12 years of age revealed 43% of seropositivity [5]. Studies conducted in Sri Lanka have focused mainly on the paediatric population and VLM. No studies have yet been carried out regarding the OLM infection status in Sri Lanka, thus the current study was carried out on 250 cases that clinicians had suspected as having ocular toxocariasis and whose serum samples had been sent for testing to determine the presence of *Toxocara* antibodies and to describe demographic factors and clinical manifestations of seropositive patients.

## Methods

### Study setting and population

A retrospective descriptive study was carried out on all the clinical samples that were referred to the Department of Parasitology, Faculty of Medicine, University of Peradeniya for serological diagnosis of toxocariasis. Serum samples were sent along with a referral letter from ophthalmology clinics of various regions of the country. Consultant Ophthalmologists clinically diagnosed these patients and referred to us for laboratory confirmation. All the patient files between the years 1995 to April 2015 were retrieved and the presenting complaints of all the patients were studied and categorized. Relevant clinical and laboratory data were extracted and entered in a Microsoft excel sheet. Variables included: demographic data, *Toxocara* antibody optical density (OD) result and the various clinical symptoms.

### Detection of anti- Toxocara IgG antibodies

From 1995 to 2015 April all the *Toxocara* OLM suspect cases were diagnosed via In-House TES-ELISA [5]. The microtitration plates were coated with 0.846 μg/ml *Toxocara* excretory secretory antigens and incubated at 4 °C overnight. Plates were then washed in washing buffer (PBS with 0.05% Tween 20), post coated with 100 μl PBS containing 1% bovine serum albumin and 2.5% sucrose and incubated at room temperature (RT) for one hour. The plates were washed five times with wash buffer. Subsequently 100 μl of diluted (1:100) serum samples were added to the test well in duplicates and incubated for one hour at RT. Known negative and positive sera were used as control in each plate. Following the incubation, plates were washed three times in washing buffer to remove unbound serum and 100 μl of horse radish peroxidase conjugated anti-human Ig G (Sigma Chem Co.) at a dilution of 1: 5000 was added to each well. Plates were incubated 1 h at RT. Subsequently 100 μl of the substrate, o-phenylenediamine dihydrochloride, 2 mg tablets in 3% hydrogen peroxide solution (Sigma – Aldrich, India) was added to each well. After incubating for 20 min at RT, 100 μl of 3 M H_2_ SO_4_ was added to each to stop the reaction. The optical density (OD) at 492 nm was measured with an automated ELISA reader. An OD value ≥0.2 was considered to be seropositive.

### Statistical analysis

SPSS version 19.0 (SPSS Inc., Chicago, IL, USA) was used for statistical analysis of the data. Chi-square test was performed to determine the most significant symptoms. *P* value of less than 0.05 was considered to indicate statistical significance.

## Results

### Demographic details

From January 1995 to April 2015, two hundred and fifty OLM suspected patients were identified in the patient records. Most of the patients (67 /250) were between ages 5–14 years. Age range of patients was from 1 to 79 years. Of the study sample, the majority (52.4%, 131/250) were males (Table 1).

**Table 1:** Demographic details of participants

	Gender							
	Male		Female		Unspecified		Total	
Age group	n	%	n	%	n	%	N	%
0 to 4	7	5.2	10	9.1			17	6.8
5 to 9	19	14.1	7	6.4	2	40	28	11.2
10 to 14	20	14.8	19	17.3			39	15.6
15 to 19	16	11.9	9	8.2			25	10
20 to 24	8	5.9	7	6.4			15	6
25 to 29	12	8.9	11	10.0			23	9.2
30 to 34	4	3.0	4	3.6			8	3.2
35 to 39	8	5.9	6	5.5			14	5.6
40 to 44	11	8.1	12	10.9			23	9.2
45 to 49	4	3.0	8	7.3			12	4.8
50 to 54	8	5.9	4	3.6			12	4.8
55 to 59	7	5.2	3	2.7			10	4
60 to 64	2	1.5	0	0.0			2	0.8
65 to 69	2	1.5	3	2.7			5	2
70 to 74	2	1.5	1	0.9			3	1.2
75 to 79	1	0.7	0	0.0			1	0.4
Unknown	4	3.0	6	5.5	3	60	13	5.2
Total	135		110		5		250	

*Detection of anti- Toxocara IgG antibodies.*

When tested with an In-House TES-ELISA 62% of the cases (*n* = 155) showed an OD value ≥0.2 indicating seropositivity. The age range of the seropositive patients was from 1 to 78 years with the mean age of 27 years. The highest number of seropositivity (25/155) was observed within the age group of 10 to 14 years. Males showed higher seropositivity than that of females (Table 2).

**Table 2:** Prevalence of seropositive males and females in each age group

	Seropositive males		Seropositive female		Total	
Age group	N	%	n	%	N	%
0 to 4	4	4.5	3	4.5	7	4.5
5 to 9	14	15.7	6	9.1	20	12.9
10 to 14	12	13.5	13	19.7	25	16.1
15 to 19	10	11.2	6	9.1	16	10.3
20 to 24	5	5.6	4	6.1	9	5.8
25 to 29	10	11.2	5	7.6	15	9.7
30 to 34	2	2.2	1	1.5	3	1.9
35 to 39	4	4.5	4	6.1	8	5.2
40 to 44	8	9.0	6	9.1	14	9.0
45 to 49	3	3.4	6	9.1	9	5.8
50 to 54	6	6.7	3	4.5	9	5.8
55 to 59	5	5.6	3	4.5	8	5.2
60 to 64	0	0.0	0	0.0	0	0.0
65 to 69	1	1.1	2	3.0	3	1.9
70 to 74	2	2.2	0	0.0	2	1.3
75 to 79	1	1.1	0	0.0	1	0.6
Unknown	2	2.2	4	6.1	6	3.9
Total	89		66		155	

### Clinical manifestations and Seropositivity

The most frequent clinical presentation of seropositive OLM cases were uveitis (34.19%) followed by reduced vision (21.94%), vitritis (12.9%) and choroiditis (7.74%) (Table 3). Of these clinical manifestations only vitritis was significantly associated with TES-ELISA seropositivity (X^2^ = 8.557, *p* = 0.003) (Table 4).

**Table 3:** Percentage of seronegative and seropositive patients with clinical manifestation

	ELISA positive (*n* = 155)		ELISA negative (*n* = 95)		Total (*n* = 250)	
Symptom	Number	%	Number	%	Number	%
Retinal leision	10	6.45	3	3.16	13	5.2
Choroiditis	12	7.74	9	9.47	21	8.4
Uveitis	53	34.19	35	36.84	88	35.2
Vitritis	20	12.90	2	2.11	22	8.8
Endophthalmitis	3	1.94	2	2.11	5	2
Reduced vision	34	21.94	18	18.95	52	20.8
Retinal detachment	5	3.23	1	1.05	6	2.4
Granuloma	15	9.68	7	7.37	22	8.8
Macular scarring	7	4.52	9	9.47	16	6.4
Macular coloboma	1	0.65	1	1.05	2	0.8
Band keratoma	1	0.65	0	0.00	1	0.4
Exudative Retinopathy	1	0.65	0	0.00	1	0.4
Macular oedema	2	1.29	0	0.00	2	0.8

**Table 4:** Chi Square analysis

Symptom	Chi square	*p* Value
Retinal leision	1.2962	0.255
Choroiditis	0.2296	0.632
Uveitis	0.1811	0.67
Vitritis	8.557	0.003
Endophthalmitis	0.0087	0.926
Reduced vision	0.3192	0.572
Retinal detachment	1.1875	0.276
Granuloma	0.3913	0.532
Macular scarring	2.4165	0.12
Macular coloboma	0.1232	0.726
Band keratoma	0.6154	0.433
Exudative retinopathy	0.6154	0.433
Macular oedema	1.2357	0.266

*P* value ≤0.05 was considered to indicate statistical significance

## Discussion

Seroepidemiological surveys, done in various countries of the world indicate that larva migrans due to *Toxocara* spp. is a relatively common infection in the general population and at times it can lead to serious or life threatening conditions in human [16]. Clinical features associated with toxocariasis are common and non-specific. Therefore, diagnosis of toxocariasis based solely on the clinical findings is unreliable. Since the *Toxocara l*arva fails to complete their migratory cycle in the human, eggs are not passed in the stool. Also, the definitive diagnosis of toxocariasis by histological examination for *Toxocara* larvae in biopsy material is very difficult. Thus, the confirmatory diagnosis of toxocariasis depends heavily on immunological tests. The successful in vitro culture of *T. canis* larvae has enabled the collection of excretory secretory antigens of the second stage larva [17] and introduction of ELISA based on these antigens [18]. The lack of sensitivity and specificity associated with many of the early serodiagnostic techniques were rectified, resulting in considerable improvement in immunodiagnosis. A seroepidemiological study on toxocariasis carried out in children aged 1–12 years in Sri Lanka has reported 43% of seropositivity, indicating high level of transmission. This is the first study on ocular toxocariasis carried out in a Sri Lankan population. The present study showed considerably high *Toxocara* IgG antibody carriage (62%) among clinically suspected OLM patients. This pattern of antibody carriage is much higher than the prevalence of 21% and 28% reported among clinically suspected OLM patients reported in North India [19] and Slovenia [20] respectively. In Sri Lanka, although rabies is a high public health priority little attention is paid to other zoonotic diseases that humans could acquire from dogs. A survey in an urban area has shown dog to human population ratio of 1:8 [21] with 20% of these dogs being un-owned or group as strays. Thus the risk of soil contamination with *Toxocara* spp. is an increasing threat throughout human habitations in Sri Lanka. This could be the reason for our community to have high seroprevalence rate compared to prevalence rate reported elsewhere in the world.

The present study showed high prevalence of OLM among children aged between 10 and 14 years. This is in contrast to a study published in 2009, where the infection was most frequently seen in children of ages below 10 years [22]. The current study observed high incidence of OLM in males. Similarly, several prior studies have documented that OLM is more common among males [23, 24].

The most common symptoms and signs include reduced vision, peripheral posterior pole retinal granuloma, tearing, leukokoria and redness of the eye. Only a single eye was affected in most of the patients which is in line with a study done to determine the worldwide seroprevalence of OLM [25]. The major cause of visual acuity loss is uveitis (34.2%). However only vitritis (*P* = 0.003) was significantly associated with seropositivity. A larger case-controlled study is required to study the ocular manifestations in seropositive cases. A prior study reported vitritis, cystoid macular oedema and tractional retinal detachment as the major causes of visual acuity loss.However, in the present study, only a small proportion of the patients harbored macular oedema (1.29%) or retinal detachment (3.23%) [26]. Although the exact reason for the clinical presentation is not known the most possible reason may be due to a toxic or immunoallergic reaction against the larva antigens which leads to inflammatory reactions and granuloma formation. An immunological response to highly immunogenic antigens is considered to cause vitritis.

Diagnosis was performed by In-house TES-ELISA specific to *Toxocara canis* coupled with the clinical features observed in the patient. In the current study 34.2% of the seronegative cases presented with uveitis while 7/22 granuloma cases were seronegative. Toxoplasmosis and tuberculosis are possible differential diagnosis for uveitis and granuloma. However, we could not perform any laboratory investigations to exclude these diseases in our study. One limitation of this study was that clinicians have not specified the type of granulomas and uveitis. Posterior pole granuloma (27) and posterior uveitis (11) are shown to be frequent clinical presentations of OLM. Those seronegative cases could be due to some other reasons. It has been reported that the diagnosis of ocular toxocariasis is challenging since the levels of antibodies in serum may be low or undetectable [27]. Hence even a low level of antibody maybe of diagnostic value, but the cut-off value for such an instance has not been established so far [25]. Sensitivity of *Toxocara* ELISA can be improved by analyzing intraocular fluid [28]. In such cases it is worthwhile to obtain vitreous or aqueous humor fluid to perform ELISA. However, even the use of these fluids cannot be 100% accurate since it was observed that those who had clear signs of OLM did not show seropositivity in the eye fluid. Therefore it is advisable to detect antibodies by ELISA in both serum and eye fluid to enhance the sensitivity of detecting OLM [29].

Our study had a few limitations that need further consideration. This was a retrospective descriptive study. *Toxocara* antibodies can be positive among healthy people; however there is no data for the prevalence of seropositivity of *T.canis* in asymptomatic individuals. Therefore a case control study comparing seropositive rates in healthy, asymptomatic patients and those with ocular manifestations of ocular toxocariasis is required. Also, after laboratory diagnosis, we were unable to conduct a follow up on the individuals after treatment, since they were sent to us only for serological testing. Further studies with proper serological follow up with treatment are essential to establish proper treatment in our country.

## Conclusion

In conclusion, the results of this study showed high seroprevalence of toxocariasis among clinically suspected OLM cases confirming the *Toxocara* etiology. This high rate of *Toxocara* seropositivity in ocular patients should alert ophthalmologists in Sri Lanka to include toxocariasis in the differential diagnosis of ocular diseases presented with the symptoms and signs stated above. This neglected tropical disease must be recognized and preventive measures must be reinforced in the country to control canine toxocariasis.

## Abbreviations

OLM: Ocular larva migrans
VLM: Visceral larva migrans
TES-ELISA: *Toxocara* excretory – secretory antigen enzyme linked immunosorbent assay
ELISA: Enzyme linked immunosorbent assay
NLM: Neurological toxocarisis
OD: Optical density
PBS: Phosphate buffered saline
RT: Room temperature
SPSS: Statistical Package for the Social Sciences
